# Infants with cystic fibrosis have altered fecal functional capacities with potential clinical and metabolic consequences

**DOI:** 10.1186/s12866-021-02305-z

**Published:** 2021-09-15

**Authors:** Alexander Eng, Hillary S. Hayden, Christopher E. Pope, Mitchell J. Brittnacher, Anh T. Vo, Eli J. Weiss, Kyle R. Hager, Daniel H. Leung, Sonya L. Heltshe, Daniel Raftery, Samuel I. Miller, Lucas R. Hoffman, Elhanan Borenstein

**Affiliations:** 1 Department of Genome Sciences, University of Washington, Seattle, WA USA; 2 Department of Microbiology, University of Washington, Seattle, WA USA; 3 Department of Pediatrics, University of Washington, Seattle, WA USA; 4 Division of Gastroenterology, Hepatology, and Nutrition, Department of Pediatrics, Baylor College of Medicine, Houston, TX USA; 5 Cystic Fibrosis Foundation Therapeutics Development Network Coordinating Center, Seattle Children′s Research Institute, Seattle, WA USA; 6 Northwest Metabolomics Research Center, Department of Anesthesiology and Pain Medicine, University of Washington, Seattle, WA USA; 7 Department of Medicine, University of Washington, Seattle, WA USA; 8 Pulmonary and Sleep Medicine, Seattle Children’s Hospital, Seattle, WA USA; 9 Blavatnik School of Computer Science, Tel Aviv University, Tel Aviv, Israel; 10 Department of Clinical Microbiology and Immunology, Sackler Faculty of Medicine, Tel Aviv University, Tel Aviv, Israel; 11 Santa Fe Institute, Santa Fe, NM USA

**Keywords:** Cystic fibrosis, Fecal microbiome, Metagenomics, Metabolomics, Infants, Nutrition

## Abstract

**Background:**

Infants with cystic fibrosis (CF) suffer from gastrointestinal (GI) complications, including pancreatic insufficiency and intestinal inflammation, which have been associated with impaired nutrition and growth. Recent evidence identified altered fecal microbiota taxonomic compositions in infants with CF relative to healthy infants that were characterized by differences in the abundances of taxa associated with GI health and nutrition. Furthermore, these taxonomic differences were more pronounced in low length infants with CF, suggesting a potential link to linear growth failure. We hypothesized that these differences would entail shifts in the microbiome’s functional capacities that could contribute to inflammation and nutritional failure in infants with CF.

**Results:**

To test this hypothesis, we compared fecal microbial metagenomic content between healthy infants and infants with CF, supplemented with an analysis of fecal metabolomes in infants with CF. We identified notable differences in CF fecal microbial functional capacities, including metabolic and environmental response functions, compared to healthy infants that intensified during the first year of life. A machine learning-based longitudinal metagenomic age analysis of healthy and CF fecal metagenomic functional profiles further demonstrated that these differences are characterized by a CF-associated delay in the development of these functional capacities. Moreover, we found metagenomic differences in functions related to metabolism among infants with CF that were associated with diet and antibiotic exposure, and identified several taxa as potential drivers of these functional differences. An integrated metagenomic and metabolomic analysis further revealed that abundances of several fecal GI metabolites important for nutrient absorption, including three bile acids, correlated with specific microbes in infants with CF.

**Conclusions:**

Our results highlight several metagenomic and metabolomic factors, including bile acids and other microbial metabolites, that may impact nutrition, growth, and GI health in infants with CF. These factors could serve as promising avenues for novel microbiome-based therapeutics to improve health outcomes in these infants.

**Supplementary Information:**

The online version contains supplementary material available at 10.1186/s12866-021-02305-z.

## Background

Cystic fibrosis (CF) is a genetic disorder that impacts multiple organs, including the lungs, pancreas, liver, gut, sweat gland, and reproductive system [18]. CF is caused by mutations in the CF transmembrane conductance regulator (CFTR), a protein that regulates ion and fluid transport. Impaired CFTR function leads to abnormal hydration and pH at epithelial cell membranes, resulting in altered mucus constituency and clearance at mucosal surfaces. CF gastrointestinal (GI) disease often manifests early in life with pancreatic exocrine insufficiency (PI), which results in nutrient malabsorption, intestinal obstruction, and intestinal inflammation [7, 34].

The resulting altered CF GI milieu likely plays a role in shaping differences in the fecal microbiota observed in people with CF compared to those with healthy GI tracts. The gut microbiota normally play several important roles in host health, including energy harvest [3], nutrient synthesis [26], bile acid metabolism [47], and resistance to pathogen infection [10]. However, fecal microbiome studies have shown that the CF and non-CF GI microbiomes differ in both taxonomic composition [17, 49] and functional capacities encoded by the microbiota [21]. This CF dysbiosis manifests early in childhood [42], with abnormal bacterial species and functional gene abundances that are associated with increased fecal measures of inflammation and dietary fat malabsorption [28, 35]. These manifestations of CF GI dysfunction may result from abnormal GI microbial community activities, as suggested by the altered fecal metabolite profiles observed among children with CF [60]. Therefore, the CF GI microbiome may contribute to disease pathogenesis, and therapies that target or address the functional consequences of this dysbiosis could improve GI health, growth, and development among infants with CF and other causes of nutrient malabsorption.

In a recent study, we characterized the CF gut dysbiosis during the first year of life and examined associations between taxonomic changes in the gut microbiota and body growth in infants with CF [27]. We found differences in gut microbiota composition between healthy infants and infants with CF as early as 4 months of age; several of these disparities grew more pronounced over time, including reduced prevalences of bacteria capable of producing anti-inflammatory short-chain fatty acids (SCFAs) [61]. Additionally, we demonstrated that infants with CF exhibited a significant delay in fecal microbiota development during the first year of life when compared to normal infants. The CF infant gut dysbiosis, and delay in microbiota development, were most marked among infants with short stature, suggesting a potential effect of the gut microbiota on linear growth in CF, consistent with emerging evidence for a role of GI microbiota functions in early bone and body growth [63].

During the first year of life, healthy infants exhibit dynamic changes in their fecal microbial taxonomic composition [4]. Some of these shifts are concurrent with dietary changes, such as cessation of breast-feeding and increase in food variety, suggesting the importance of diet in selecting the microbiota. In turn, these taxonomic changes are predicted to result in changing functional properties of the microbiota that impact infant GI development, including production of amino acids and vitamins. In our previous study, we identified significant delays in taxonomic development of the infant CF fecal microbiota relative to healthy infants, but it was unclear what the microbial functional consequences, if any, of this delayed development might be. Specifically, functional redundancy between microbial species [40] and strain-level variation in genomic content [24] render the link between taxonomic differences and functional differences extremely complex [19, 38], and it is possible, for example, that functional differences may be less pronounced than observed taxonomic differences and may not reflect delayed development in infants with CF. Furthermore, it is uncertain how the influences of diet or other external factors on the GI microbiota, as observed previously in healthy infants [4], might translate to infants with CF given the altered environment of the CF GI tract. Considering what is known about healthy infant GI microbial functional development, we hypothesized that the altered composition and development of the CF infant fecal microbiota would result in altered functions critical for host nutrition and GI health.

To test this hypothesis, we performed functional characterization of the CF infant gut microbiome. Using functionally annotated shotgun metagenomic sequencing data, we identified a significant delay in the development of CF GI microbiota functional capacities that was apparent by 6 months of age. We also found several functional differences between infants with CF who were breast-fed compared to those who were formula-fed, as well as between infants with and without concurrent antibiotic treatment, suggesting that these dietary and treatment factors play important roles in shaping CF GI microbiome function and could accordingly serve as potential avenues for therapeutic intervention. Finally, we detected several metabolites important for nutrient absorption with abundances that correlated with those of specific fecal microbes at 12 months, suggesting potential targets for improving the nutritional outcomes of infants with CF and other causes of malnutrition.

## Results

### Gut microbiota functional capacities diverge between healthy and CF infants during the first year of life

**Fig. 1:**
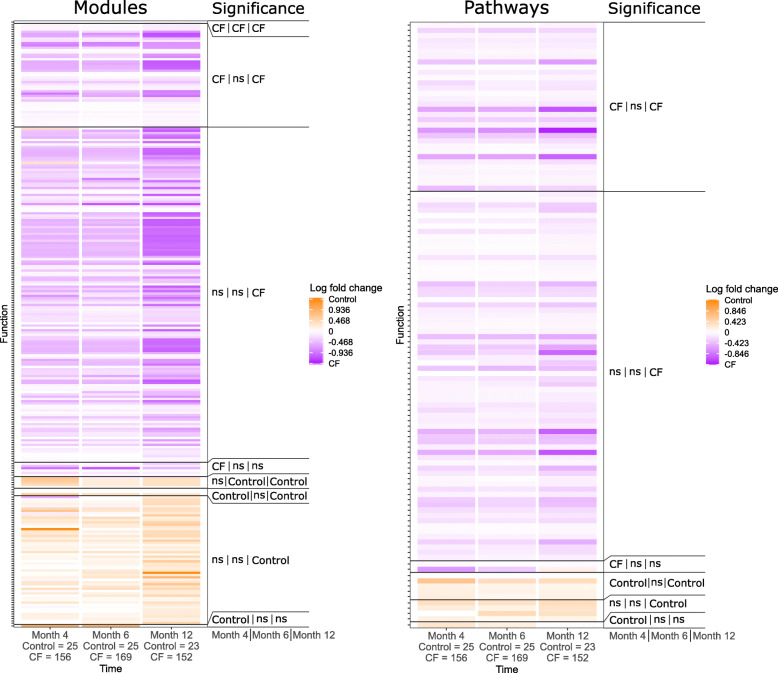
Differential abundances of modules and pathways between healthy infants and infants with CF during the first year of life. Each cell in the heatmap represents the log_10_ ratio of the median abundances of controls to CF. Modules/pathways are grouped vertically by temporal pattern of significant differential abundance (*q* < 0.01, Wilcoxon rank-sum test). Significance labels indicate at which time points and in which cohort the function was significantly increased in one cohort relative to the other (CF for significantly increased in infants with CF, Control for significantly increased in healthy controls, and ns for not significant). Sample sizes at each time point are indicated in the x-axis label

To compare fecal microbiota functional capacities between healthy controls and infants with CF during the first year of life, we performed shotgun metagenomic sequencing on 1157 fecal samples collected longitudinally from 207 infants with CF and from 122 samples collected from 25 healthy controls. We annotated this metagenomic data using MetaLAFFA [20] to quantify the abundances of functional modules and pathways according to the Kyoto Encyclopedia of Genes and Genomes (KEGG) database [30] (see Methods). We then compared the resulting fecal metagenomic-based functional profiles between the two cohorts at each shared time point. By 4 months of age, 56 modules and 40 pathways exhibited significantly different abundances (*q* < 0.01, Wilcoxon rank-sum) between infants with (*n* = 207) and without CF (*n* = 25), and by 12 months these differences were even more pronounced, with 256 modules and 111 pathways displaying significantly different abundances (*q* < 0.01, Wilcoxon rank-sum, Fig. 1 and Supp. Table 1). Functional categories that differed among the two infant groups were highly consistent between months 4 and 12, with 49 modules and 37 pathways differentially abundant at both time points and no changes in directionality when differences were significant. Additionally, among these consistent modules and pathways, the fold change increased from month 4 to month 12 in 36 (out of 49) modules and 27 (out of 37) pathways, indicating that the difference in functional capacities expands during the first year of life.

Interestingly, we found that many of the functions observed to be significantly different in abundance at months 4 and 12 were not so at month 6, with only 6 modules differentially abundant at month 6 (*q* < 0.01, Wilcoxon rank-sum, Fig. 1 and Supp. Table 1), which is commonly a period of marked transition in the infant diet [23], including increased solid food intake [11]. However, among functions with nonsignificant differences at month 6, directionality remained highly consistent with observed differences at months 4 and 12. In other words, the majority of modules and pathways differentially abundant at either month 4 or month 12 exhibited similar patterns at month 6, and the decreased magnitudes of some differences at 6 months could be attributable to the common dietary transition shared among both cohorts.

To further explore the differences in fecal functional metagenomic capacities between these two cohorts, we next focused on those classes of modules and pathways that were significantly differentially abundant between cohorts at month 12, when differences were generally most pronounced. The functional categories exhibiting large differences (ratio > 2x) included two-component regulatory systems (33 modules; e.g. BaeS-BaeR, BasS-BasR, and EnvZ-OmpR), biosynthetic capabilities (24 modules, 7 pathways; e.g. lipopolysaccharide biosynthesis, betaine biosynthesis, GABA biosyntehsis), and transport systems (43 modules; e.g. maltose/maltodextrin transport system, glycine betaine/proline transport system, iron complex transport system; Supp. Table 1). Notably, CF-associated modules were enriched for functions related to antibiotic resistance compared to control-associated modules (*p* < 0.01; Chi-Squared test), and the only resistance modules relatively increased in controls were associated with vancomycin resistance. We also observed that pathways encoding metabolism of the SCFAs butyrate and propionate were significantly more abundant in CF samples relative to controls at months 4 and 12. Furthermore, consistent with our previous work [35], the differences between CF and control samples, measured as the log ratio of median pathway abundances, between butyrate and propionate metabolism pathways decreased during the first year of life. These SCFA metabolism pathways represented 2 of only 16 pathways with diminishing differences between CF and control samples over time, compared to the 27 pathways with increasing differences over the same time interval.

### Infants with CF display delayed development of fecal microbiota functional capacities

We previously showed that infants with CF displayed a delay in the development of their fecal microbiota compared to controls [27]. Given this delayed pattern of change in microbial species constituency, and considering the functional metagenomic differences we identified above, we hypothesized that the functional differences observed in the CF infant fecal microbiome represent a developmental delay relative to healthy controls during the first year of life, rather than a general or random disruption of fecal microbial community functions.

**Fig. 2:**
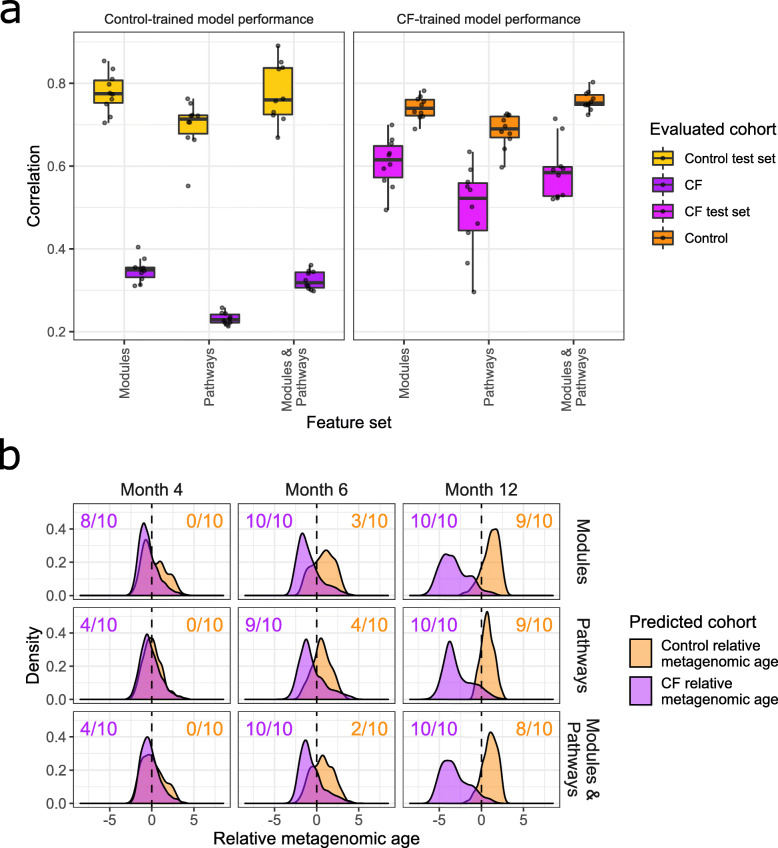
Metagenomic age model performance and relative metagenomic age predictions. (**a**) Boxplots displaying performance of metagenomic age models across 10 trained replicate models trained on 3 different sets of functional features. Left panel shows correlations between true and predicted ages for predictions made using model replicates trained on a subset of healthy infants, right panel shows correlations using model replicates trained on a subset of infants with CF. Color indicates which cohort the model is generating age predictions for. (**b**) Densities of relative metagenomic age predictions made on one cohort using models trained on the opposite cohort (CF ages predicted by control-trained models, control ages predicted by CF-trained models). Fractions on the left indicate the number of replicate models that predicted relative metagenomic ages significantly below 0 for CF, fractions on the right indicate relative metagenomic ages significantly shifted above 0 for controls

To investigate this possibility, we adapted a technique known as microbiota age analysis [53] to define the developmental patterns over time in the functional capacities of healthy control fecal metagenomes, and determine whether this development was delayed in infants with CF. Briefly, we first trained Random Forest models to predict host age based on associated metagenomic functional profiles for our cohort of healthy controls (see Methods). To assess the predictive power of these models, we trained each model 10 times, each time using 70% of the control samples for training and measuring its accuracy using the withheld 30% of samples. This assessment demonstrated that the trained metagenomic age models incorporating both module and pathway abundances successfully predicted control samples’ ages (*q* < 0.01; 0.67 < *r* < 0.89; Pearson correlation; Fig. 2a), indicating a notable age-dependent trend in functional capacities consistent with previous findings on normal gut microbiota functional development during infancy [4]. Models trained on the metagenomes of infants with CF used a similar 70–30% sample split for training and predictive power assessment. The CF-trained models also displayed notable predictive power on the 30% reserved CF samples when utilizing module and pathway data (*q* < 0.01; 0.52 < *r* < 0.71; Pearson correlation; Fig. 2a), indicating an age-dependent pattern of development of CF fecal microbial functional capacity similar to the pattern observed in controls.

We next used metagenomic age models trained on one cohort to predict the host age of infants from the opposite cohort. Similar to our previous study defining fecal microbiota age [27], differences between true and predicted host metagenomes are referred to here as “relative metagenomic ages” and indicate potential functional differences in the developmental process between the two cohorts. For example, if a model trained on healthy controls and applied to a CF infant sample were to predict the CF host age to be younger than the true age, that CF sample would be said to reflect a negative relative metagenomic age, and accordingly *delayed* metagenomic development relative to healthy controls. Using this definition, we found that the fecal samples from infants with CF displayed nonsignificant trends toward delayed development in metagenomic functional capacities starting at month 4, increasing in magnitude over time, resulting in significant delays by month 12 (Fig. 2b). This delay in CF metagenomic development was mirrored by relatively accelerated fecal metagenomic development (positive relative metagenomic age) in healthy controls compared to CF (Fig. 2a,b).

### Clinical and dietary differences among infants with CF are associated with differences in fecal metagenomic functional capacities

**Fig. 3:**
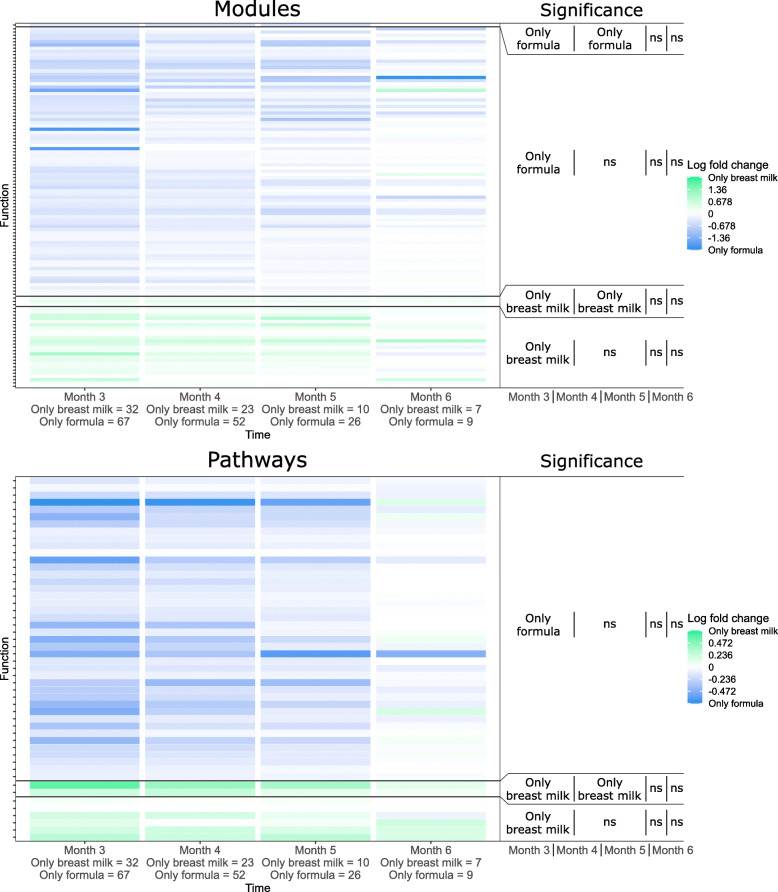
Differential abundances of modules and pathways between exclusively breast-fed and exclusively formula-fed infants with CF during the first year of life. Each cell in the heatmap represents the log_10_ ratio of the median abundances of breast-fed to formula fed. Modules/pathways are grouped vertically by temporal pattern of significant differential abundance (*q* < 0.01, Wilcoxon rank-sum test). Significance labels indicate at which time points and in which cohort a specific function was significantly increased in one cohort relative to the other (Only formula for significantly increased in formula feeding infants relative to only breast milk fed infants, Only breast milk for significantly increased in breast feeding relative to formula feeding infants, and ns for not significant). Sample sizes at each time point are indicated in the x-axis label

The above results revealed significant functional fecal metagenomic differences between healthy controls and infants with CF, suggesting that manipulating the CF microbiome towards a healthier functional composition could be beneficial. Such efforts require a deeper understanding of the factors, both external and innate, that shape the functional content of the CF fecal microbiome, which in turn could reveal potential modulators. To identify these factors, we compared functional profiles between infants with CF who differed in various dietary and clinical variables shown previously to be associated with altered microbiome composition, including diet (breast-fed versus formula-fed, pre- versus post-solid food introduction, [11, 23]), antibiotics usage (naïve versus prior history, on versus off at time of sampling, [22]), and growth status (stunted versus non-stunted linear growth, [27]). We focused this portion of the analysis on the CF cohort because complete information on diet and antibiotics were unavailable for the healthy controls. Our analysis revealed significant differences in functional capacities for two comparisons: infants solely breast-fed compared with those solely formula-fed, and infants receiving versus not receiving antibiotics at the time of sampling. Specifically, significant differences in functional capacities in infants exclusively breast-fed versus those formula-fed (*q* < 0.01, Wilcoxon rank-sum test) included 112 modules and 50 pathways at month 3, and 4 modules and 2 pathways significantly different at month 4 (Fig. 3). The differentially abundant functional categories included 31 modules and 35 pathways related to biosynthesis, degradation, and metabolism, and 45 modules and 1 pathway related to molecular transport. Among metabolism-related categories, the majority (22 modules and 30 pathways) were increased in formula feeding-only infants relative to breast feeding-only infants, while molecular transport categories showed no specific general association with either dietary condition (Supp. Table 2). These differences in metabolic and transport functional capacities are consistent with previous reports of functional differences among breast- and formula-fed healthy infants [4], though we could not confirm this in our healthy cohort due to small samples sizes for these dietary groups among our healthy controls. Specifically, when we analyzed the metagenomic data from the Bäckhed study using the same approach as with our CF cohort, we found 21 modules and 4 pathways to be associated with the same dietary condition at 4 months of age in both studies (*q* < 0.05, Wilcoxon rank-sum test, Supp. Table 3). These results suggest that diet has a similar impact on the functional capacities of the microbiota in the CF and healthy infant GI tract, though at the module level the observed differences tended to be smaller in magnitude in infants with CF (*q* < 0.1, Wilcoxon rank-sum test). By comparison, the influence of antibiotics on CF fecal metagenomic content appeared to occur later in infancy. Concurrent antibiotic usage was associated with decreased fecal abundances of diverse modules and pathways relative to infants not taking antibiotics at month 8 (*q* < 0.01, Wilcoxon rank-sum test, Supp. Table 4), but not in any other time points.

**Fig. 4:**
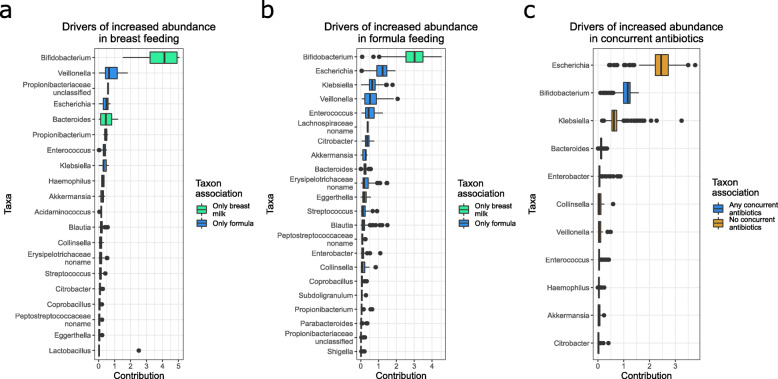
Boxplots of FishTaco contributions to driving function differential abundances between cohorts within CF. Genera are ordered based on their median contribution to driving a function’s differential abundance across all modules/pathways found differentially abundant in (**a**) infants at month 3 that were solely breast feeding, (**b**) infants at month 3 that were solely formula feeding, and (**c**) infants at month 8 on antibiotics. Only genera with at least one contribution greater than 0.1 are displayed. Color indicates in which cohort the genus’s relative abundance was higher

These functional differences among subgroups of infants with CF could contribute to differences in clinical outcomes and are therefore candidates for targeted intervention. To inform identification of potential interventions, we sought to identify the taxa that drove these functional differences, focusing on functional variation among dietary and antibiotic usage conditions. To identify taxonomic drivers of functional differences, we used FishTaco [38], a permutation-based method that quantifies the relationships between shifts in taxonomic and functional features (see Methods). This analysis identified several genera, *Escherichia, Bifidobacterium, Klebsiella*, *Veillonella, Enterococcus, Bacteroides*, and an unclassified genus in the Lachnospiraceae family, as top predicted drivers of functional differences between exclusively breast-fed and formula-fed infants with CF at month 3 (Fig. 4a, b, Supp. Table 5). The role of *Bifidobacterium* as a primary contributor to diet-dependent differences in infant fecal functional capacities is consistent with previous observations of relatively increased fecal abundances of *Bifidobacterium* in breastfed infants [4, 52] and their known capacities for metabolizing glycans that differ between formula and breast milk [58]. Interestingly, three of these genera, *Escherichia, Bifidobacterium,* and *Klebsiella,* were also predicted to be primary drivers of functional differences in infants currently on versus off antibiotics at month 8 (Fig. 4c, Supp. Table 5). Given the well-described importance of *Bifidobacterium* in the infant gut [2], we further examined species-level effects on our findings within this genus and found *B. breve* to be the primary contributor to functional differences between dietary conditions within this genus, whereas *B. longum* was the more relevant species when comparing antibiotics usage (Supp. Figure 1).

Given these diet- and antibiotics-associated differences, we wanted to confirm that the differences we observed between healthy infants and infants with CF were not driven by dietary differences or increased antibiotics usage in CF infants, but are rather inherent to the CF condition. To this end, we compared functional metagenomic content between healthy infants and exclusively breast-fed CF infants, exclusively formula-fed CF infants, CF infants with no concurrent antibiotics usage, and CF infants with no prior antibiotics usage. It is worth noting that introduction of solid foods and antibiotics usage information were not available for our healthy infants, so we were unable to completely match healthy and CF cohorts based on dietary or antibiotics usage conditions. We found that the functional differences between healthy and CF infants described above were mostly independent of these dietary and antibiotics usage factors. Specifically, 83% of the functions differentially abundant at month 4 between healthy and CF infants were also differentially abundant when comparing healthy and exclusively formula-fed CF infants at month 4 (Supp. Table 6). Similarly, 73% were consistent when comparing healthy and CF infants with no concurrent antibiotics usage, and 79% were consistent when comparing healthy and CF infants with no prior antibiotics usage. At month 12, we found that 98% of the differentially abundant functions between healthy and CF infants were also differentially abundant when only comparing healthy infants to CF infants with no concurrent antibiotics usage, and 70% were also differentially abundant when only considering CF infants with no prior antibiotics usage. Interestingly, there were no differentially abundant functions when comparing healthy infants and exclusively breast-fed CF infants at month 4, which suggests that formula feeding in CF infants may drive early functional differences. However, when we compared healthy infants at month 12 with CF infants at month 12 who had been exclusively breast-fed prior to the introduction of solid foods, we still found that 59% of the functions reported above remained differentially abundant.

The observed metagenomic differences between our dietary and antibiotics usage CF cohorts suggested a potential impact of these factors on the development of fecal metagenomic functional capacities. However, when we implemented a metagenomic age analysis similar to our comparison of healthy and CF relative metagenomic age, we found that metagenomic age models trained on these cohorts performed very poorly (75% of replicate models with *r* < 0.38, Pearson correlation, on withheld test sets), rendering downstream age analysis on these cohorts unreliable.

### Variation in fecal metabolite abundances is consistent with predicted microbial influences

The observed variation in encoded functional capacities among CF infant fecal microbiomes suggests that the metabolic activities of, and the metabolites produced by, the GI microbiota differ among these infants. To investigate this possibility, we measured aqueous metabolites from the fecal samples of 84 infants with CF at month 12.

**Fig. 5:**
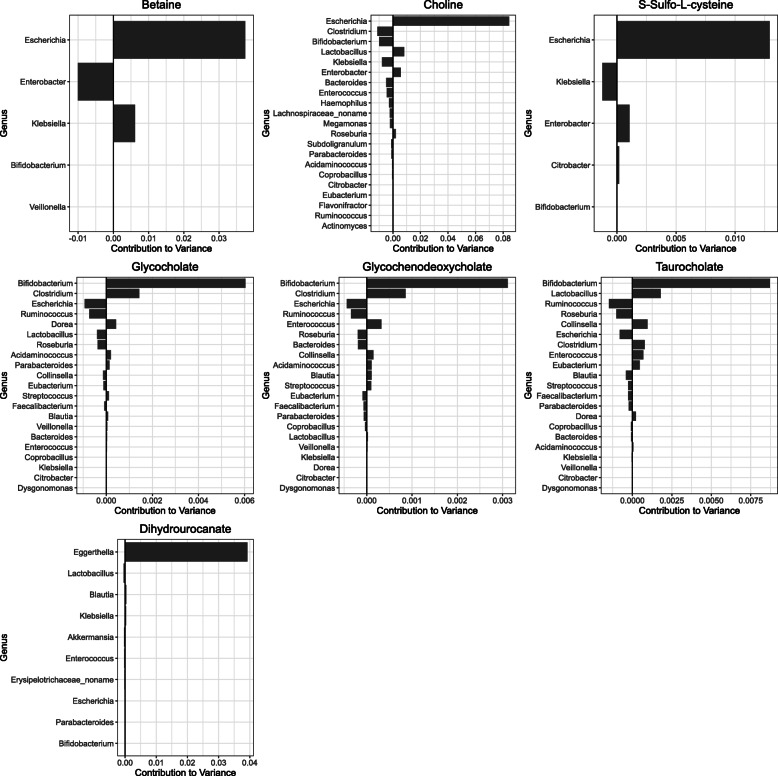
Quantified taxonomic contributors to the variance of aqueous metabolite abundances. Each bar indicates the MIMOSA2-calculated contribution of a genus to the variance in a metabolite’s abundance. Genera are ordered vertically by the absolute value of their contribution

In contrast to our functional metagenomic analysis, we identified no significant differences in metabolite abundances among the CF cohort associated with dietary features or antibiotic exposure (*q* < 0.05, Wilcoxon rank-sum test), and we were unable to examine differences between our healthy and CF cohorts due to lack of metabolomics data for our healthy cohort. However, it remained possible that variation in microbial metabolic activities across the study group independent of these two variables could impact abundances of metabolites important to host physiology, representing candidate targets for intervention. To identify microbial taxa potentially involved in cohort-wide variation in fecal metabolite abundances (as opposed to abundance differences between two cohorts), we used MIMOSA2 [43], which quantifies microbial contributions to variation (within a cohort) in measured metabolite abundances using genome-informed mechanistic models of microbial metabolism (see Methods). MIMOSA2 identified 7 metabolites in our dataset exhibiting variation in fecal abundance that was well-explained by the associated taxonomic profiles and models of microbial metabolism (*q* < 0.05): betaine, choline, glycocholate, glycochenodeoxycholate, taurocholate, dihydrourocanate, and S-sulfo-L-cysteine (Fig. 5), suggesting that fecal abundances of these metabolites are partially governed by the metabolic activity of the gut microbiota. Reassuringly, many of the taxa that MIMOSA2 identified as contributing to the variance of these metabolites were also identified above as drivers of differences in fecal microbiota functional capacities. Specifically, *Escherichia* was the largest microbial contributor to variance in betaine, choline, and S-sulfo-L-cysteine abundances, along with contributions from *Enterobacter, Klebsiella, Bifidobacterium, Clostridium*, and *Lactobacillus* (Fig. 5, Supp. Table 7). The bile acids glycocholate, glycochenodeoxycholate, and taurocholate shared *Bifidobacterium* as the largest contributor to variation, along with contributions from *Clostridium*, *Escherichia, Ruminococcus, Lactobacillus*, and *Roseburia*. These genera were linked to bile acid variation by encoding genes for choloylglycine hydrolase, which degrades these bile acids. Unlike the other metabolites, a single genus, *Eggerthella,* was responsible for most of the microbe-associated variation of dihydrourocanate, with only minor contributions from other genera. When we examined species-level contributions to metabolite variance within the *Bifidobacterium* genus, we again found notable species-level variation in their contributions to measured levels of choline and the three bile acids mentioned above (Supp. Figure 2). In particular, *B. bifidum* was the primary contributor for choline, whereas *B. longum*, *B. adolescentis*, *B. pseudocatenulatum*, and *B. catenulatum* were the primary *Bifidobacterium* species contributing to bile acid variation and in the same direction as the contribution of the genus as a whole. The identification of *Escherichia, Bifidobacterium,* and *Klebsiella* as both contributors to variation in metabolites and drivers of differences in functional capacities suggests that they may play an important role in shaping the overall function of the gut microbiome in infants with CF during the first year of life.

## Discussion

Consistent with our original hypothesis, we found that fecal microbiota functional capacities in healthy infants and infants with CF diverge during the first year of life. By 12 months of age, the resulting differences included multiple modules and pathways related to bacterial metabolism and interaction with the environment. We determined that this functional divergence indicated a significant delay in the development of fecal microbiota functional capacities that normally occurs during the first year of life. We also found that exclusive breast or formula feeding and concurrent antibiotic usage had noticeable effects on fecal microbiota functional capacities in infants with CF. Finally, using fecal metabolomic data from a subset of the infants with CF at 12 months of age, we identified several metabolites, including bile acids, amino acid derivatives, and other potentially clinically-relevant molecules discussed below, that exhibited fecal abundances determined to be regulated in part by the microbiota based on cohort-wide variation in microbial and metabolite abundances. These findings pinpoint microbial metabolic pathways and metabolites that, if found to be of potential relevance for infant growth, nutrition, and development, could be modulated via the microbiota for novel interventional targets.

The observed relative delay in CF fecal microbiota functional development may indicate that CF gut microbiota cannot provide the normal benefits of a healthy gut microbiome during infancy [3, 10, 26]. By 12 months of age, the CF-related delayed functional development was characterized by differential abundances of genes involved in bacterial environmental responses, metabolism, and antibiotic resistance, suggesting potential impairment of these capabilities. For example, CF-associated differences in metabolic gene abundances and their associated functions could contribute to the growth failure that is common among infants with CF, which we recently showed to be associated with the infant CF taxonomic dysbiosis [27]. While we did not detect any significant associations between differences in specific functional capacities during the first year of life and linear growth failure, our analysis identified other predicted functional impairments and metabolomic differences among infants with CF that could potentially contribute to early nutritional outcomes that we did not measure.

Specifically, we identified several bacterial taxa with notable contributions to variation in both functional capacities and metabolite abundances within the CF infant cohort. For example, our results indicate that *Bifidobacterium* was both a driver of differences in functional capacities between breast-fed and formula-fed infants and the largest quantified contributor to variation in bile acid abundances. Bile acids are involved in several aspects of gut function, including lipid digestion, nutrient absorption, and metabolic regulation [1], and microbial metabolism of bile acids is an important component of bile acid activity [62]. While there is controversy about whether bile acid absorption in the CF GI tract is impaired in people with CF [44], studies have shown that the absorption of taurocholate and glycocholate is not intrinsically altered by CFTR dysfunction [57]. Furthermore, work in mouse models suggests that CFTR dysfunction can lead to the microbiota-mediated impairment of farnesoid X receptor signaling in ileal epithelial cells, a key component of bile acid metabolism regulation in the gut [29]. These findings, and the significant correlation we identified in CF samples between these bile acid abundances and those of specific bacterial genera (such as *Bifidobacterium, Escherichia,* and *Clostridium spp.*) known to modify these moieties [59], suggest that the differential fecal abundances of these molecules we observed are likely influenced by the activities of gut microbes, as suggested previously for people with CF [46]. Bile acid homeostasis plays important roles in nutrient absorption and protection against pathogens such as *C. difficile*, and altered bile acid homeostasis is thought to be involved in a wide range of CF-associated metabolic abnormalities, including control of lipid and glucose regulation as well as liver and intestinal disease (reviewed in [46]).

Our results also identified other metabolites, including choline and dihydrourocanate, that were likely modulated at least in part, by the GI microbiota, and previous work has indicated that these metabolites have potential links to CF disease. For example, plasma levels of choline have been shown to be relatively low in CF patients and to correlate directly with lung function and indirectly with serum markers of inflammation [25]. More generally, choline has been recognized as an important factor in neurodevelopment, and low choline intake has been associated with diverse conditions, including heart disease, inflammation, and breast cancer [65]. Dihydrourocanate, which is produced by bacteria including *Eggerthella*, is known to impair glucose tolerance and insulin signaling, common problems among young adults with CF [31]. While no specific links to CF disease are evident for betaine or S-sulfo-L-cysteine, both are known substrates for bacterial metabolism. Betaine is synthesized by diverse bacteria by metabolizing choline or glycine, and is a cofactor in diverse metabolic activities by bacteria and the host alike [67]. S-sulfo-L-cysteine has been shown to be a specific product of gram-negative bacterial enzymes [41, 45]; its role in human health and disease is not clear. Therefore, our results highlight effects of the infant CF GI dysbiosis on metabolites of both known and unknown clinical significance.

These notable differences in functional capacities and variation in microbe-associated abundances of metabolites linked to CF suggest that microbiome-targeted therapeutics could offer potential benefits in ameliorating GI symptoms of CF. Regarding possible candidates for future therapeutic avenues, our results indicate that, even in the altered physicochemical environment of the CF gut, antibiotics and diet play the same important roles in shaping the fecal microbiome as has been shown in healthy infants [4, 48]. Antibiotics are used frequently to treat the chronic lung infections common among people with CF, and antibiotic exposure during infancy has been shown to have lasting effects on the GI antibiotic resistome [22]. Here, we found that antibiotic treatment was associated with functional metagenomic differences driven largely by species known to be either selected by (*Escherichia* and *Klebsiella* spp.), or inhibited by (*Bifidobacterium* spp.), antibiotics in the human GI tract [22, 48], supporting the substantial impact of these medications on the CF GI microbiome. It should also be noted that due to the frequency of antibiotics usage in infants with CF, we were unable to reasonably exclude infants that had previously received antibiotics from this and other comparisons made in this study. While this may be a residual confounding factor in our results, when we performed our analyses of CF infants stratified by prior antibiotics usage, we found no notable metagenomic differences.

Similar to the effect of antibiotics, we found that functional capacities of the CF fecal microbiota were affected by diet- specifically, by whether infants were exclusively breast versus formula-fed, as also seen in healthy infants [4]. The notable effects of diet could explain why we observed relatively few significant functional differences between healthy and CF infants at 6 months of age, which is commonly a period of important dietary transitions such as weaning from milk and introduction of solid foods in both healthy and CF infants [6, 51], as indeed observed in our CF cohort (Supp. Table 8). Regardless of the cause of this period of functional similarity, the effect appeared transient, as CF-associated differences were even more pronounced by 12 months of age than before 6 months. The relatively large impact of diet on GI microbial functional capacity evident from these findings raises the possibility of developing effective dietary interventions to influence infant GI microbiota functions, including but even beyond children with CF.

We identified associations between CF, diet, fecal microbial functional capacities, and fecal metabolite profiles, suggesting likely contributors to infant GI function that, if validated, represent candidate biomarkers of a variety of clinically-relevant outcomes in CF and targets for therapy. For example, our results suggest that dietary or probiotic approaches targeting abundances or functions of specific taxa in the GI tract could improve diverse health outcomes in infants with CF and related disorders. The associations we identified between microbes and specific fecal metabolites may also indicate which microbes are likely to contribute to the fecal abundances of those metabolites in CF. This information, combined with additional data on links between metabolite abundances and health outcomes, can help us determine how we might use microbial abundances as a tool for driving changes in the infant CF fecal metabolome to approach a more desirable state. For example, modulating the intestinal levels of *Bifidobacterium* spp., *Clostridium* spp., or *Escherichia coli* would be predicted to affect bile acid abundances in the CF infant gut, potentially improving nutritional, intestinal, and hepatic outcomes. Given the clear effects of diet, and particularly milk-associated glycans, on *Bifidobacterium* abundances, supplementing or depleting specific nutrients in the infant diet may be sufficient to achieve these goals. More specifically, targeted efforts to increase the abundances of *Bifidobacterium* species, and in particular *B. longum,* could increase bile acid deconjugation and thereby improve colonic reabsorption [59] and CF bile acid homeostasis [46]. Several animal models of CF have been shown to recapitulate features of human CF intestinal disease and dysbiosis [39, 54], providing a convenient system for testing microbiome-targeting interventions.

Regarding potential tools and methods for future therapeutics, probiotics could serve as a cost-effective approach for targeted manipulations, and there have already been several trials of probiotic usage in CF [13], some of which have incorporated *Bifidobacterium* species. However, none of these trials have investigated the impact of probiotics on bile acids. *B. longum*, specifically, seems like a promising candidate for probiotics aimed at improving CF bile acid homeostasis, due to its broad impact on several bile acids that we observed. Given the potentially undesirable shift in taxonomic and functional composition of the CF gut microbiome due to antibiotics usage, fecal microbiota transplants (FMTs) could also play a role in future therapeutics. FMTs have shown success in counteracting the negative GI consequences of antibiotics usage [14, 55] and have been effective in modulating the infant gut microbiome [32]. However, to date there has only been one reported case of FMT in a patient with CF, to treat a *Clostridium difficile* infection [16], and further work is required to understand how such techniques might be applied to treat CF GI symptoms.

## Conclusions

Our results clearly demonstrate that the fecal metagenome is altered in infants with CF, consistent with our previous finding concerning differences in the taxonomic composition of the infant microbiome in CF. The delayed development of fecal functional capacities related to GI health in CF identified by our analysis suggests that the infant gut microbiome could play a role in early nutrition and growth failure in infants with CF. We also identified functional variation in the fecal metagenomic content among CF infants associated with differences in diet and antibiotic usage, indicating the potential for manipulation of the CF infant GI metagenome via these, or similar, tools. Furthermore, our integrated analysis of taxonomic and metabolomic data revealed several important GI metabolites, including three bile acids, that were correlated with taxonomic abundances in CF. Combined, these results suggest several potential avenues for the development of microbiome-based therapeutics for improving health outcomes, in particular nutrition and growth, in infants with CF.

## Methods

### Sample collection

Sample collection details have been described previously [27] and are replicated here for convenience.

Fecal samples were collected from 207 of the 231 infants who participated in the BONUS study, a longitudinal, observational cohort study that was conducted during regularly scheduled CF clinic visits in the first year of life at 28 US Cystic Fibrosis Foundation-accredited Care Centers in the CF Foundation Therapeutic Development Network. The primary goal of BONUS was to examine incremental weight gain, linear growth and clinical features in the first year of life in infants with CF who underwent newborn screening. Detailed study design and methods, including descriptions of consent for the samples obtained and institutional review board approvals, have been published previously [33]. Fecal samples were collected at 3, 4, 5, 6, 8, 10 and 12 months of age (1157 total samples). Stool was uniformly collected at home in sterile collection cups provided by the study within 1 d of each clinic visit and kept at 2–8 °C until frozen at − 70 °C in the clinic.

Fecal samples were collected from 25 healthy infants as part of a prospective, single-center, observational cohort study to follow healthy infants not affected with CF for the first year of life. Written informed consent was obtained from all parents or guardians of the study participants, and institutional review board approval was provided by the University of Buffalo. Fecal samples were collected at 2, 4, 6, 9 and 12 months of age (122 total samples); therefore, age matching for the two cohorts was possible for the 4, 6, and 12 month time points. Stool was collected in study-provided sterile containers at home and kept at − 20 °C until delivered to the clinic. Although this storage protocol differs from that of the CF cohort, there is extensive literature demonstrating that storage at 4 °C versus − 20 °C during the time period of this study generates very similar taxonomic profiles of fecal samples [5, 12, 56].

### Dietary and clinical metadata collection

Detailed diet data were recorded for CF subjects and included presence/absence of breast milk, formula, and table food in diet (Supp. Table 8). Antibiotics usage data were also collected and included class of antibiotic, age (in days) when treatment began, and age (in days) when treatment ended or if treatment was ongoing at the end of the study. To standardize comparisons, we coded antibiotic usage data as a binary feature indicating whether any antibiotics had been taken at any time prior to sample collection or not and several similar binary features encoding the same data for individual classes of antibiotics. Introduction of solid foods and antibiotics data were unavailable for healthy subjects.

### Metagenomic sequencing and functional annotation

Detailed DNA extraction and sequencing protocols have been described previously for this dataset [27] and are replicated here for convenience.

The conduct of this ancillary study was judged to be exempt from review as human subjects research by the University of Washington Human Subjects Division Institutional Review Board. DNA extraction from fecal samples has been described previously [28, 64]. The resulting DNA samples were stored at − 80 °C until sequencing. For each sample, a random-fragment library was constructed using the Nextera DNA Sample Preparation Kit (Illumina) with dual indexing and sequenced on the HiSeq 2500 platform to produce 96-bp paired-end reads. Sequencing generated an average of 29.6 million reads per sample, and 97% of samples had > 10 million reads. Initial FASTQ files were filtered prior to subsequent analysis. Human DNA sequence was identified and removed using KneadData 0.35 (http://huttenhower.sph.harvard.edu/kneaddata) with the Hg-19 human reference genome. Reads were filtered and trimmed for quality using Trimmomatic 0.33 (http://www.usadellab.org/cms/index.php?page=trimmomatic). Duplicate reads were removed using EstimateLibraryComplexity, part of the Picard Tools package (https://broadinstitute.github.io/picard/), and the Sequniq 0.1 Python package (https://github.com/standage/sequniq). All software packages were run using default settings.

Functional profiles were obtained using MetaLAFFA [20] in its default configuration. Briefly, reads were aligned to the KEGG [30] database of microbial genes (2019) using DIAMOND [9] on standard sensitivity settings and an e-value cutoff of 0.001. The best match (or best matches in the case of ties) based on e-value were kept for each read and the rest of the alignments were discarded. Gene counts were calculated by summing up the number of reads that mapped to each gene. When a read matched equally well to multiple genes, that count was distributed fractionally and evenly among those genes. Gene counts were converted to KEGG Orthology (KO) counts based on the functional annotations associated with those genes in the KEGG database. When a gene was associated with multiple KOs, its count was fractionally and evenly distributed among those KOs. We then normalized KO abundances using MUSiCC [36]. These normalized KO abundances were then aggregated to KEGG module and pathway abundances using the support-based approach from EMPANADA [37].

### Targeted MRM metabolomics

Fecal samples were extracted using methanol , frozen at − 80 °C and then transported on dry ice to the UW Northwest Metabolomics Research Center (NW-MRC) for analysis. Briefly, targeted aqueous metabolite profiling analysis was performed using a Sciex 6500+ liquid chromatography tandem mass spectrometry (LC-MS/MS) instrument (Sciex, Toronto Canada) and standard operating procedures developed previously [50, 66]. The LC-MS/MS analysis is based on hydrophilic interaction chromatography (HILIC) using a Waters XBridge BEH Amide column (150 mm × 2.1 mm, 2.5 μm; Waters Corporation, Milford, MA). The platform targeted 308 metabolites that are located in more than 50 different metabolic pathways. This assay provides detailed information on metabolites involved in glycolysis, tricarboxylic acid cycle (TCA), pentose phosphate shunt, as well as amino acid, fatty acid, bile acid and nucleic acid metabolism and other pathways. Twenty-six isotope labeled internal standard were included to both monitor sample preparation steps and system performance.

Each sample was injected twice, 2 μL and 10 μL, for analysis using positive and negative ionization modes, respectively. Both chromatographic separations were performed in HILIC mode with a flow rate of 0.3 mL min-1, autosampler temperature set to 4 °C, and the column compartment set at 40 °C. The mobile phase was composed of Solvents A (5 mM ammonium acetate in H2O + 0.5% acetic acid + 0.5% acetonitrile) and B (acetonitrile + 0.5% acetic acid + 0.5% water). The LC gradient conditions were the same for both positive and negative ionization modes. After an initial 1.5 min isocratic elution of 10% A, the percentage of Solvent A was increased linearly to 65% at t = 9 min. Then the percentage of A remained the same (65%) for 5 min (t = 14 min), and the percentage of A was reduced to 10% at t = 15 min to prepare for the next injection. The total experimental time for each injection was 30 mins. MS ionization was performed using an electrospray ionization (ESI) source and peak integration was performed using Sciex Analyst software. A pooled study sample was used as the QC and run once for every 10 study samples. The intra-assay median CV based on this QC sample was 3.9% across all the study.

### Metagenomic age analysis

The metagenomic age analysis was adapted from the concept of microbiota age analysis [53] and followed the same methodology to our prior work, for which detailed protocols have been described previously [27] and are replicated here for convenience with minor adjustments indicating the use of module- and pathway-level functional data in model creation.

Metagenomic age was determined using an approach similar to that of Subramanian et al. [53]. To train a model, control samples were randomly subsampled to achieve the same number of samples (23) per time point. These samples were then randomly split into a training set and a testing set (for model evaluation) using a 70–30% split while guaranteeing that time points were equally represented in the training and testing sets. This resulted in 80 total training samples and 35 total testing samples, with 16 training samples per time point and 7 testing samples per time point within the training and testing sets. Regularized random forests [8, 15] were then fitted to the training set (R package RRF, ntree = 10,000, all other parameters set to the default). To assess whether model performance was noticeably affected by choice of training and testing samples, this process was performed ten times to produce ten replicate models, each trained and evaluated on different randomly selected training and testing sets. This process, including training ten replicate models, was repeated for different feature sets including all module abundances, all pathway abundances, and all module and pathway abundances together. Models fitted to CF data were trained and evaluated in a similar manner. Owing to the small number of CF samples available at month 2, the numbers of training and testing samples were similar at each time point (20 training samples, 9 testing samples per time point), although the total number of training and testing samples was larger because more time points were available for infants with CF (160 training samples and 72 testing samples).

Model performance was evaluated based on the Pearson correlation between model predictions for sample metagenomic age and the true age of the sample. Correlations were calculated separately for each replicate model using either the testing set associated with the replicate or the full set of CF samples. Relative metagenomic age was calculated by comparing predicted metagenomic age to a spline fitted to the associated model. For each replicate model, a spline was fitted using model predictions on the associated testing set as a function of the true age of the test samples (R function smooth.spline, d.f. = 3, all other parameters set to the default). A sample’s relative metagenomic age was then defined as a model’s prediction of the sample’s metagenomic age minus the value of the model’s associated spline at the true age of the sample. Each sample had ten estimates of its relative metagenomic age, one from each replicate model trained on the opposite cohort. The significance of negative or positive shifts in relative metagenomic age at each time point was calculated using the Wilcoxon signed-rank test with the null hypothesis that the median relative metagenomic age was zero for each cohort at each time point.

### Functional shift decomposition

Taxonomic contributions to functional shifts were estimated using FishTaco [38], a tool that uses a game theory-inspired permutation-based technique to decompose individual contributions to changes in a function’s abundance. FishTaco was run using the genus-level MetaPhlAn2 taxonomic profiles associated with this dataset [27], with inferred genomic content. For each function, taxonomic contributions were ranked from most positive to most negative (largest drivers to largest attenuators) independent of which cohort the genus was associated with. *Bifidobacterium* species-level contributions were calculated by separating the *Bifidobacterium* genus into its constituent species and reanalyzing the data with all other taxa left at the genus level.

### Metabolomic variation contribution analysis

MIMOSA2 [43] was used to estimate each taxon’s contribution to variation in metabolite abundances. MIMOSA2 uses functionally annotated genomic content for each taxon, combined with metabolic reaction stoichiometry, to create a mechanistic model of microbial community’s metabolism. MIMOSA2 then uses this model to determine the component of the experimentally-observed variance in metabolite abundances that can be explained by variation in the abundances of taxa that synthesize or degrade those metabolites. We reported results based on models with significant (q < 0.05) fits to the experimentally-observed data. The genus-level genomic content inferred by FishTaco was used as the basis for MIMOSA2’s mechanistic model of community metabolism. *Bifidobacterium* species-level contributions were calculated by separating the *Bifidobacterium* genus into its constituent species and reanalyzing the data with all other taxa left at the genus level.

### Statistics

Two-tailed Wilcoxon rank-sum tests were used to determine the significance of differential function abundances. Metagenomic age model prediction Pearson correlation significance was determined using two-tailed t-tests with t-value equal to (*r* * sqrt(*n* - 2))/sqrt(1 – *r*^2) and *n* – 2 degrees of freedom, where *r* was the correlation coefficient and *n* was the sample size. Significance of enrichment of differentially abundant specific functional categories was determined using a chi-squared test. MIMOSA2 model fit significances were determined using the reduction in dispersion test. All *p*-values were converted to *q*-values when multiple tests were performed using the Benjamini-Hochberg procedure.

## Supplementary Information

**Additional file 1: Supp. Figure 1**. Boxplots of FishTaco contributions to driving function differential abundances between cohorts within CF, highlighting *Bifidobacterium* species. Taxa are ordered based on their median contribution to driving a function’s differential abundance across all modules/pathways found differentially abundant in (**a**) infants at month 3 that were solely breast feeding, (**b**) infants at month 3 that were solely formula feeding, and (**c**) infants at month 8 on antibiotics. Only taxa with at least one contribution greater than 0.1 are displayed. Color indicates in which cohort the taxon’s relative abundance was higher.

**Additional file 2: Supp. Figure 2**. Quantified taxonomic contributors to the variance of aqueous metabolite abundances, highlighting *Bifidobacterium* species. Each bar indicates the MIMOSA2-calculated contribution of a taxon to the variance in a metabolite’s abundance. Taxa are ordered vertically by the absolute value of their contribution.

**Additional file 3: Supplementary Table 1**. Differentially abundant modules and pathways between CF and control infants. **Supplementary Table 2**. Differentially abundant modules and pathways between exclusively breast fed and exclusively formula fed CF infants. **Supplementary Table 3**. Differentially abundant modules and pathways between exclusively breast fed and exclusively formula fed healthy infants. **Supplementary Table 4**. Differentially abundant modules and pathways between CF infants on antibiotics and off antibiotics. **Supplementary Table 5**. FishTaco-estimated contributions of taxonomic drivers to functional shifts for within-CF dietary and antibiotics usage comparisons. **Supplementary Table 6**. Differentially abundant modules and pathways between control infants various dietary and antibiotics usage cohorts of CF infants. **Supplementary Table 7**. MIMOSA-estimated contributions of taxa to variation in CF infant fecal metabolites at month 12. **Supplementary Table 8**. Summary of infant diet and antibiotic statistics by age.

## Data Availability

All metagenomic DNA sequence data required to assess the metagenome-based conclusions of this research are available without restriction from the Sequence Read Archive at the National Center for Biotechnology Information under BioProject accession PRJNA510445. Metabolomic data is available from the corresponding authors upon reasonable request.
