# Supplementary material for: Infants with cystic fibrosis have altered fecal functional capacities with potential clinical and metabolic consequences
Source: BMC Microbiol. 2021 Sep 15;21:247. doi: 10.1186/s12866-021-02305-z (PMC8444586; doi:10.1186/s12866-021-02305-z)
Supplement: Supplementary file 1 — Additional file 1: Supp. Figure 1. Boxplots of FishTaco contributions to driving function differential abundances between cohorts within CF, highlighting Bifidobacterium species. Taxa are ordered based on their median contribution to driving a function’s differential abundance across all modules/pathways found differentially abundant in (a) infants at month 3 that were solely breast feeding, (b) infants at month 3 that were solely formula feeding, and (c) infants at month 8 on antibiotics. Only taxa with at least one contribution greater than 0.1 are displayed. Color indicates in which cohort the taxon’s relative abundance was higher. [file 12866_2021_2305_MOESM1_ESM.pdf]

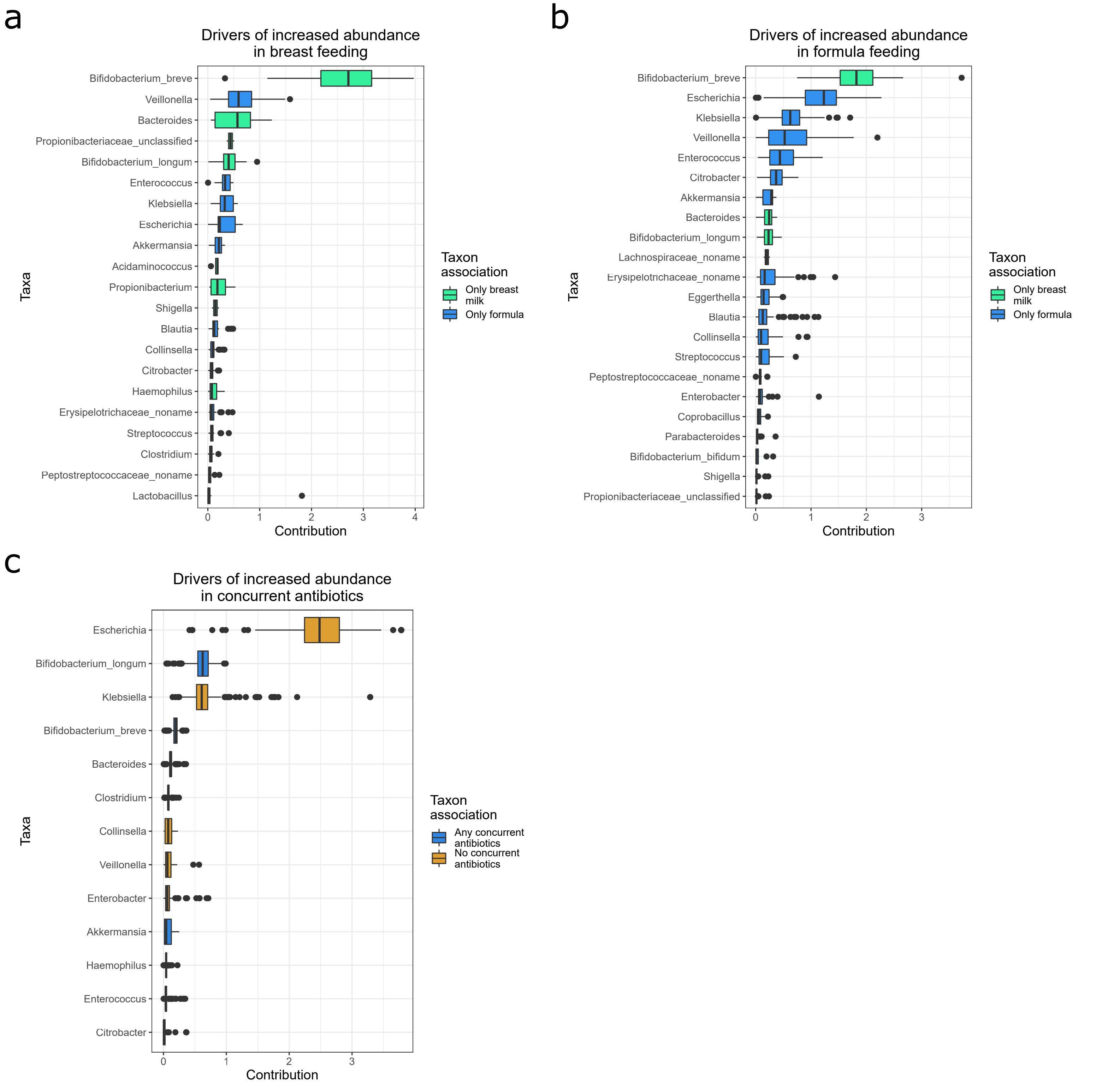

a
Drivers of increased abundance in breast feeding
Bifidobacterium_breve
Veillonella
Bacteroides
Propionibacteriaceae_unclassified
Bifidobacterium_longum
Enterococcus
Klebsiella
Escherichia
Akkermansia
Acidaminococcus
Propionibacterium
Shigella
Blautia
Collinsella
Citrobacter
Haemophilus
Erysipelotrichaceae_noname
Streptococcus
Clostridium
Peptostreptococcaceae_noname
Lactobacillus
Taxa
0
1
2
3
4
Contribution
Taxon association
Only breast milk
Only formula
b
Drivers of increased abundance in formula feeding
Bifidobacterium_breve
Escherichia
Klebsiella
Veillonella
Enterococcus
Citrobacter
Akkermansia
Bacteroides
Bifidobacterium_longum
Lachnospiraceae_noname
Erysipelotrichaceae_noname
Eggerthella
Blautia
Collinsella
Streptococcus
Peptostreptococcaceae_noname
Enterobacter
Coprobacillus
Parabacteroides
Bifidobacterium_bifidum
Shigella
Propionibacteriaceae_unclassified
Taxa
0
1
2
3
Contribution
Taxon association
Only breast milk
Only formula
c
Drivers of increased abundance in concurrent antibiotics
Escherichia
Bifidobacterium_longum
Klebsiella
Bifidobacterium_breve
Bacteroides
Clostridium
Collinsella
Veillonella
Enterobacter
Akkermansia
Haemophilus
Enterococcus
Citrobacter
Taxa
0
1
2
3
Contribution
Taxon association
Any concurrent antibiotics
No concurrent antibiotics
