# Supplementary figures and images for: Infants with cystic fibrosis have altered fecal functional capacities with potential clinical and metabolic consequences
Source: BMC Microbiol. 2021 Sep 15;21:247. doi: 10.1186/s12866-021-02305-z (PMC8444586; doi:10.1186/s12866-021-02305-z)

Choline

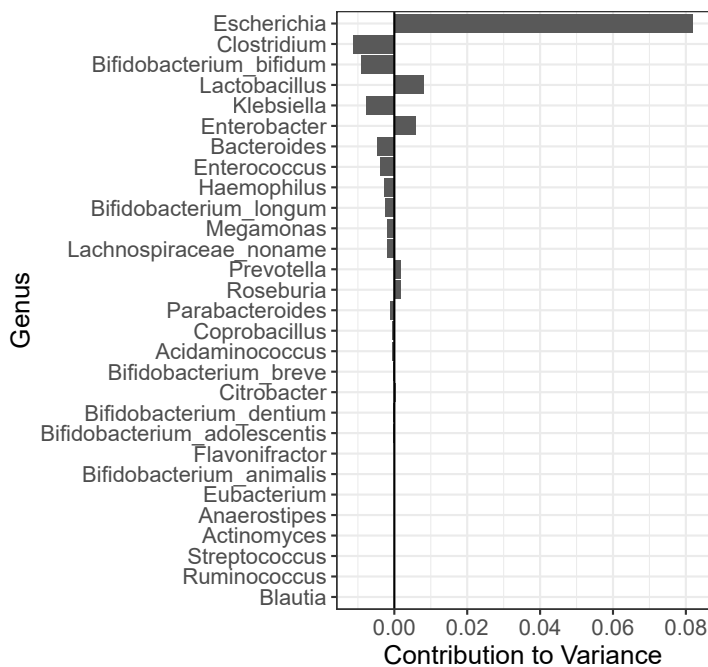

Glycocholate

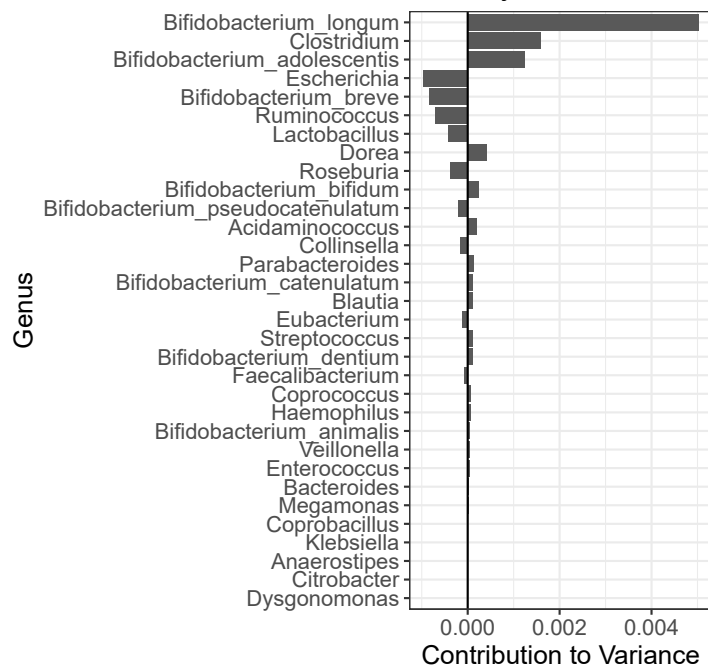

Glycochenodeoxycholate

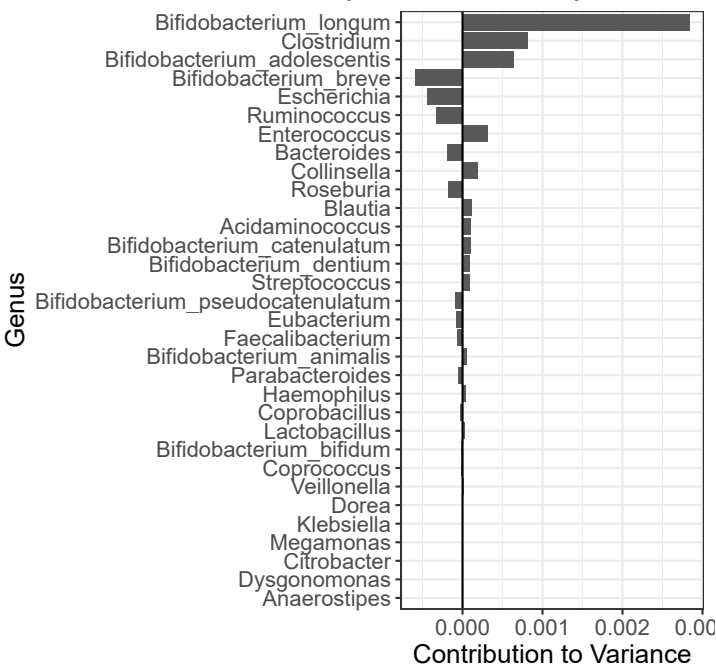

Taurocholate

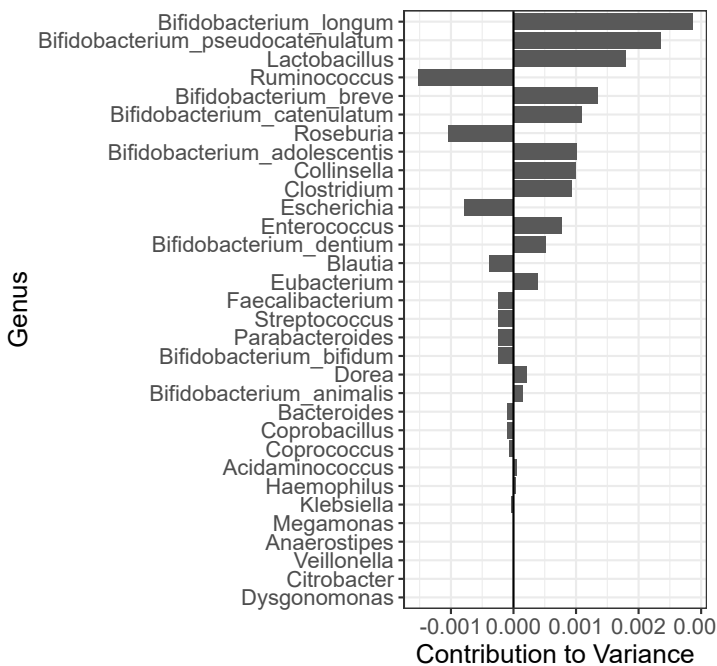

Supplement: Supplementary file 2 — Additional file 2: Supp. Figure 2. Quantified taxonomic contributors to the variance of aqueous metabolite abundances, highlighting Bifidobacterium species. Each bar indicates the MIMOSA2-calculated contribution of a taxon to the variance in a metabolite’s abundance. Taxa are ordered vertically by the absolute value of their contribution. [file 12866_2021_2305_MOESM2_ESM.pdf]
